# Climate Variability and Change and Their Potential Health Effects in Small Island States: Information for Adaptation Planning in the Health Sector

**DOI:** 10.1289/ehp.8429

**Published:** 2006-07-11

**Authors:** Kristie L. Ebi, Nancy D. Lewis, Carlos Corvalan

**Affiliations:** 1 ESS, LLC, Alexandria, Virginia, USA; 2 East-West Center, Honolulu, Hawaii, USA; 3 Department of Public Health and Environment, World Health Organization, Geneva, Switzerland

**Keywords:** adaptation, climate change, climate variability, human health, small island states, vulnerability

## Abstract

Small island states are likely the countries most vulnerable to climate variability and long-term climate change. Climate models suggest that small island states will experience warmer temperatures and changes in rainfall, soil moisture budgets, prevailing winds (speed and direction), and patterns of wave action. El Niño events likely will strengthen short-term and interannual climate variations. In addition, global mean sea level is projected to increase by 0.09–0.88 m by 2100, with variable effects on regional and local sea level. To better understand the potential human health consequences of these projected changes, a series of workshops and a conference organized by the World Health Organization, in partnership with the World Meteorological Organization and the United Nations Environment Programme, addressed the following issues: the current distribution and burden of climate-sensitive diseases in small island states, the potential future health impacts of climate variability and change, the interventions currently used to reduce the burden of climate-sensitive diseases, additional interventions that are needed to adapt to current and future health impacts, and the health implications of climate variability and change in other sectors. Information on these issues is synthesized and key recommendations are identified for improving the capacity of the health sector to anticipate and prepare for climate variability and change in small island states.

Former President Leo Falcam of the Federated States of Micronesia stated, “for Pacific Island States, climate change and its associated effects are our main security concern” (Falcam 2001). The same could be said for many small island states.

There is no standard definition of a small island state (Schmidt 2005). The Small Island Developing States Network defines them as small islands and low-lying coastal countries that share similar sustainable development challenges (Small Island Developing States Network 2003); this review includes 37 of the 51 small island developing states and territories. Islands such as the U.S. Virgin Islands and Netherlands Antilles were excluded because they are parts of other countries, and countries such as Belize were excluded because they are not islands. The discussion in this article also applies, to varying degrees, to island territories of larger nations, vulnerable coastal locations, and isolated islands in archipelagic nations such as Indonesia and the Philippines.

Small island states share many features that constrain their ability to adapt to current climate variability and future climate change, including their small or very small physical size, remoteness from major land masses, limited natural resources (often with unique animal and plant life), vulnerability to natural disasters and extreme weather events, economies sensitive to external shocks, populations with high growth rates and densities, poorly developed infrastructure, and limited financial and human resources (Nurse et al. 2001). Some islands have significant emigration and “brain drain.” Small island states also display wide diversity: the islands differ in geologic type, size, elevation, soil composition, drainage characteristics, and natural resources. Some of the larger islands have significant elevation, whereas others are low-lying small coral atolls with limited or no land for evacuation in times of natural disaster. Natural resources range from scarce to abundant. Some islands have abundant surface water, whereas others are completely dependent on groundwater; water requirements are just as diverse. Social, cultural, and economic settings also vary. Some islands have large commercial or industrial centers, and others have extensive agriculture. Infrastructure, including health infrastructure, is sometimes poorly developed. Human communities range from large densely populated cities to small villages and dispersed rural populations.

The diversity of the small island states in demographic, health, economic, environment, and climate indicators is shown in Tables 1 and 2. As shown in Table 1, populations range from 2,000 in Niue to > 11 million in Cuba, with the percentage living in urban areas ranging from 13% in Papua New Guinea to 100% in Nauru. Particularly in Asia and the Pacific, many small island states have young populations, with a significant fraction of the population younger than 15 years. Most small island states have relatively healthy life expectancies (HALEs) in the range of 50–60 years (compared with HALEs of 70 years or more in most developed countries), with approximately 7–8 years of healthy life lost in males and 9–10 years in females compared with males and females in developed countries. As shown by the probability of dying before reaching 5 years of age, most of these years of life lost are in the young. Annual growth rates during 1992–2002 ranged from negative in several small island states (in most cases, due to emigration) to > 3%. Growth rates were not associated with gross domestic product (GDP) per capita; 3% or higher growth rates were experienced in Comoros with a GDP per capita of US$278 and in Bahrain with a GDP per capita of $12,012.

Table 2 shows the diversity of small island states in environment and climate indicators. Small island states account for < 1% of global greenhouse gas emissions. Carbon dioxide (CO_2_) emissions range from just a few thousand megatons in some small island states to > 35.5 million megatons in Singapore. Energy consumption per capita has a similar broad range.

The diversity of small island states will affect both the climate change impacts they experience and their ability to adapt to these impacts. Of course, it will not be possible to adapt to some impacts, such as an island becoming uninhabitable because of sea-level rise. Current and projected climate change–related effects also will be experienced by coastal mainland areas.

To better understand the vulnerability of small island states to current climate variability and to build capacity to cope with climate change through adaptation planning, we present information in this article that is synthesized from presentations and discussions at three workshops [Samoa (July 2000), Barbados (May 2002), and the Maldives (December 2003)] and a conference (after the Barbados workshop) organized by the World Health Organization, in partnership with the World Meteorological Organization and the United Nations Environment Programme [Aron et al. 2003; World Health Organization (WHO) 2000, 2003]. Key recommendations are identified for improving the capacity of the health sector to anticipate and prepare for climate variability and change.

## Climate Variability and Change in Small Island States

### Past climatic trends

Temperatures have been increasing by as much as 0.1°C per decade in regions where small island states are located (Nurse et al. 2001). Increases in surface air temperatures have been greater than global rates of warming in areas such as the Pacific Ocean and the Caribbean Sea. Based on data from 34 stations in the Pacific from about 160° east and mostly south of the equator, surface air temperatures increased by 0.3–0.8°C during the 20th century (Nurse et al. 2001). For example, in New Caledonia and the Cook Islands, temperatures have risen 0.6–0.7°C since 1920.

Globally, average sea level rose between 0.1 and 0.2 m during the 20th century. Based on tide gauge data, the rate of global mean sea-level rise was in the range of 1.0–2.0 mm/year compared with an average rate of about 0.1–0.2 mm/year over the last 3,000 years (Nicholls and Leatherman 1996). It is difficult to establish the degree of sealevel change for individual islands because of limitations of observational records. Some observed changes consistent with global climate change include increased coastal erosion, more saline soils, shifting fishing grounds, and more droughts and water shortages (Nurse et al. 2001).

### Future climate projections

The projected area-averaged increase in surface air temperature for the 2050s is approximately 2.0°C for the Atlantic Ocean and Caribbean Sea, 2.0°C for the Pacific Ocean, 2.1°C for the Indian Ocean, and 2.8°C for the Mediterranean Sea (Nurse et al. 2001). The projected increase for the 2080s is approximately 3.1, 3.0, 3.2, and 4.3°C, respectively. Except for the Mediterranean Sea, the increases in surface air temperature are projected to be more or less uniform in both seasons. For the Mediterranean Sea, warming is projected to be greater during the summer than during the winter.

Recent trends suggest that surface temperatures in the tropical Pacific are likely to resemble more closely the warmer, El Niño, phase of the El Niño/Southern Oscillation (ENSO) cycle, with the eastern tropical Pacific projected to warm more than the western tropical Pacific (Meehl 1997; Nurse et al. 2001). This would shift rainfall eastward and could cause drought conditions over Australasia. A marginal decrease in precipitation is projected for other regions, particularly during the northern hemisphere summer, suggesting reduced water availability (Lal et al. 2002).

Models suggest that small island states will experience greater climate variability, such as more extreme high temperature and precipitation events (Nurse et al. 2001). The consequences of increased climate variability for small island states are likely to be related to changes in rainfall, soil moisture budgets, prevailing winds (speed and direction), and patterns of wave action. El Niño events will likely strengthen short-term and interannual variations. Although there is no consensus regarding the projected formation and behavior of tropical cyclones in a warmer world, there are indications that the intensity of these events may increase (Royer et al. 1998; Spennemann and Marschner 1995).

Between 1990 and 2100, global mean sea level is projected to rise by 0.09–0.88 m (Albritton and Meira Filho 2001). This will be due primarily to thermal expansion of the oceans and loss of mass from glaciers and ice caps. Sea levels are projected to continue rising for hundreds of years after stabilization of greenhouse gas concentrations because of the long time scales on which the deep ocean adjusts to climate change.

All these projections are, of course, highly uncertain. Key uncertainties include a lack of understanding of feedback processes in the climate system, lack of understanding of the probability distribution associated with temperature and sea-level rise projections under different climate scenarios, and limited capabilities of regional models.

## What Is the Current Distribution and Burden of Climate-Sensitive Diseases in Small Island States?

Many small island states currently suffer high burdens from climate-sensitive health outcomes, including morbidity and mortality from extreme weather events, certain vector-and food- and waterborne diseases.

### Extreme weather and climate events

Many small island states are particularly vulnerable to tropical cyclones, storm surges, flooding, and drought. These events can have both short-and long-term effects on human health, including drowning, injuries, increased disease transmission, decreases in agricultural productivity, and an increased incidence of common mental disorders (Hajat et al. 2003). Because these potential impacts are complex and far-reaching, the true health burden is rarely appreciated. Droughts and floods also can affect health through deterioration of water quality and quantity. Tropical cyclones, especially during or after a drought, also may predispose islands to wildfires, with their associated health consequences.

Extreme weather events due to the ENSO cycle can affect population health through the associated droughts, floods, heat waves, and disruptions in food production (Glantz 1996). The impacts of El Niño events vary geographically; for example, during an El Niño year, storm tracks shift to the west in the Pacific and tropical cyclones are 2.6 times more likely to occur near the Marshall Islands than during a regular year (Spennemann and Marschner 1995). Precipitation patterns may change during the ENSO cycle. For example, the western Pacific islands may be drier during an El Niño event, whereas the eastern Pacific islands may expect more rain than usual (Ropelewski and Halpert 1987). This pattern can vary from one El Niño event to another and over the course of an event, suggesting some of the difficulties in the practical application of climate forecast information in public health and other sectors. The weather changes experienced during El Niño events may provide clues to the environmental and health impacts that may occur with long-term climate change.

One example of the impacts of extreme events is the health consequences of the 1997–1998 El Niño on Pacific nations. In June 1997 the Pacific ENSO Application Center alerted governments that a strong El Niño was developing, that changes in rainfall and storm patterns could be expected, that severe droughts could occur as early as December, and that some islands were at unusually high risk of typhoons and hurricanes (Hamnett et al. 1998). In fact the region did experience extreme drought as well as several severe storms. For example, in Palau and on Pohnpei in the Federated States of Micronesia, water was available only 2 hr a day at the height of the drought. Even with the advanced warning, the affected nations sustained substantial agricultural losses in the form of damaged lands and ecosystems, damaged crops, and increased financial costs of importing water and food.

Decreases in water availability and agricultural production were the main causes of adverse health outcomes during the 1997–1998 El Niño event (Hamnett et al. 1998). However, the successes of the interventions launched, such as public education and awareness campaigns designed to reduce the risk of waterborne diarrheal diseases and vectorborne diseases, limited some of the resulting disease burdens. For example, despite the water shortage in Pohnpei, fewer children than normal were admitted to hospitals with severe diarrheal disease; this was attributed to frequent public health messages about water safety. Conversely, micronutrient deficiencies were found in pregnant women in Fiji, especially in regions where the drought was most extreme. The Indonesian island of Sumatra experienced massive forest fires caused by El Niño–driven droughts, affecting air quality in regions beyond the immediate burn areas and resulting in more cases than normal of respiratory illnesses and allergy symptoms.

The Caribbean also experiences adverse health effects from extreme weather events such as hurricanes, tropical storms, flooding, droughts, and, in Trinidad, localized tornadoes (WHO 2003). In 2001–2002 there were > 50 deaths in storms and hurricanes in the Caribbean and millions of dollars of losses in agriculture. For example, in May and September 2002, Jamaica experienced major flooding, resulting in four deaths, relocation of 725 persons, and infrastructure damages worth US$1 million (WHO 2003). Threats to health posed by extreme weather events in the Caribbean include insect- and rodent-borne diseases, such as dengue, leptospirosis, malaria, and yellow fever; waterborne diseases, including schistosomiasis, cryptosporidiosis, and cholera; foodborne diseases, including diarrheal diseases, food poisoning, salmonellosis, and typhoid; respiratory diseases, including asthma, bronchitis, and respiratory allergies and infections; heat-related illnesses, including sunstroke, sunburn, heat stress, heat exhaustion, and dehydration; malnutrition resulting from disturbance in food production or distribution; and anxiety and stress. A recent study by the Caribbean Epidemiology Centre and the Water and Sewage Authority of Trinidad and Tobago found that 18.6% of samples of potable water taken after heavy rainfall events were positive for *Cryptosporidium* (WHO 2003).

Large portions of the Caribbean population are not served by sewage-collection systems but rather depend on individual systems such as septic tanks, soakaways, and pit latrines. In rural communities, the practice of dumping waste in rivers, streams, and ravines is widespread. In times of high rainfall and flooding, stormwater runoff and floodwaters may become contaminated with fecal waste from these systems and can pose serious health risks. This waste can also exacerbate the effects of flooding. One of the reasons advanced for the massive flooding in Castries, St. Lucia, after Tropical Storm Debbie in 1994 was the clogging of waterways with waste (WHO 2003).

### Vectorborne and waterborne diseases

The distribution and abundance of many vectors may be affected by even small changes in ambient temperature and precipitation, or by changes in vegetation, host populations, and water availability, especially at the margins of vector distribution. Many small island states lie in tropical or subtropical zones with weather conducive to the transmission of diseases such as malaria, dengue, filariasis, and schistosomiasis. The rates of many of these diseases are increasing in small island states for a number of reasons, including poor public health practices, inadequate infrastructure, poor waste management practices, increasing global travel, and changing climatic conditions (WHO 2002). In addition to weather and climate factors, social aspects such as culture and traditions are important in disease prevalence.

High-priority diseases of concern for small island states include malaria, dengue, diarrheal disease/typhoid, heat stress, skin diseases, and acute respiratory infections and asthma. Outbreaks of these diseases can be costly in lives and economic impacts. An outbreak of dengue fever in Fiji coincided with the 1997–1998 El Niño; out of a population of approximately 856,000 people, 24,000 were affected, with 13 deaths (World Bank 2000). Although dengue is endemic in Fiji and other islands in the Pacific, large outbreaks do not occur yearly. Increased temperature during the El Niño may have facilitated mosquito population growth. The epidemic cost US$3–6 million. Neighboring islands also were affected.

## What Are the Potential Future Health Impacts of Climate Change in Small Island States?

Increasing global average temperature, sea-level rise, and extremes in the hydrologic cycle can have negative impacts on health. Primary concerns for many small island states include that climate change could change the frequency and severity of extreme weather and climate events such as cyclones, floods, and droughts, and change the range and prevalence of climate-sensitive diseases, particularly vectorborne diseases. High-priority diseases identified in the workshops include malaria, dengue fever, diarrheal diseases, heat stress, skin diseases, acute respiratory infections, and asthma. Small island states also face health-related problems due to sea-level rise, including coastal flooding; exacerbated storm surges; damaged coastal infrastructure; salination of island fresh water; damage to coastal ecosystems, coral reefs, and coastal fisheries; and population displacement.

Few studies have been conducted of the potential future health impacts of climate change under different climate change scenarios. One study by the South Pacific Regional Environment Programme estimated that the cost to Fiji of ignoring the potential impacts of climate change would be US$5–19 million by 2050 in terms of loss of public safety, increased vector- and waterborne diseases, and increased malnutrition from food shortages during extreme events, but not including direct damage from cyclones (WHO 2003). This study input projected changes in global mean temperature and sea-level rise under different climate change scenarios into a regional climate model to project regional changes, which were then input into health models to estimate impacts, from which costs were estimated.

The potential effects of climate change on dengue in the Caribbean were projected using the CIMSiM/DENSiM models (CIMSiM is a dynamic life-table simulation entomologic model of various parameters that influence the growth and reproduction of the mosquito vector for dengue fever, and DENSiM is essentially the corresponding account of the dynamics of a human population; these models account for the development of the virus within individuals and its passage between vector and human populations.) and HadCM3 (a general circulation model used to make climate projections on the basis of input from the scenarios) climate projections (WHO 2003). Projected changes in temperature and other weather variables were input into the infection model to estimate how the prevalence of dengue could change under different scenarios. Results suggested that the temperature-enhanced aspects of the system would be roughly balanced by attenuation because of reduced numbers of larval breeding sites. The bottom line was that dengue will unlikely be influenced positively or negatively by climate change in the locations examined. The dengue situation in the Caribbean in coming decades will principally reflect the success of vector control programs and infrastructure changes.

## What Interventions Are Being Used to Reduce the Current Burden of Climate-Sensitive Diseases?

Small island states have designed and implemented a variety of strategies, policies, and measures to reduce the current burden of climate-sensitive diseases, as highlighted in the following examples. Many of these initiatives recognize that the potential health impacts of climate variability and change do not need to be addressed individually; health outcomes with common risk factors, such as malnutrition and diarrheal diseases associated with the dry season, may be reduced together by the implementation of appropriate interventions.

Most of these initiatives include the development of early-warning systems to enhance opportunities for disease control. Early-warning systems typically are based on models developed using weather/climate forecasts, environmental observations, and epidemiologic data and are designed to provide warnings when increased risks could be expected. When increased risks are projected, interventions are undertaken to reduce the possible burden of disease. Evaluation and feedback are then used to improve the models. Many small island states need to build the capacity to develop integrated climate and health early-warning systems. In addition it will be important to build institutional, human, and scientific capacity for flexible and responsive action.

A national strategy for drought disaster planning was developed in Fiji during the 1997–1998 El Niño (WHO 2003). First, the drought had to be defined and perceived as a threat. A vulnerability assessment was conducted so that problems could be anticipated and resources could be concentrated where most needed. Development of a national strategy was hampered by the lack of information about the past effects of droughts; these effects may be difficult to recognize because of the slow onset of drought and a tendency for impacts to linger into subsequent years.

Although Indonesia has no national early-warning system for disease outbreaks, the Indonesian Ministry of Health advises all governors to prepare for possible outbreaks of dengue before the rainy season. Every local government carries out regular health-education campaigns, urging people to clean up breeding sites for mosquitoes, and hospitals to collect data on the incidence of dengue.

## What Additional Interventions Are Needed to Adapt to Current and Future Health Impacts?

Interventions for reducing the health impacts of climate variability include effective health education programs, improvement of health care infrastructure, disaster preparedness plans, vector monitoring and control, and appropriate sewage and solid-waste management practices. The ability to predict climate variations on a seasonal or interannual scale presents communities with the opportunity to develop the capacity and expertise to deal with climate variability, which will also help communities prepare for the effects of climate change. The range of interventions is large; the following are a few examples.

To manage the potential health impacts of extreme weather and climate events in the Caribbean, the Caricom Caribbean Environmental Health Institute recommends establishing monitoring and surveillance systems, creating an enabling environment, strengthening the public health infrastructure, promoting research, and promoting awareness and education. Adaptation options for Cuba include continuation of environmental educational plans, improvement of water systems, strengthening of surveillance systems, design of infrastructure to reduce impacts, strengthening of vaccination programs, and further understanding of the associations between climate variability and health.

Strategies for adaptation to sea-level rise fall into three main categories: retreat, accommodate, and protect. Retreat indicates the planned abandonment of land to reduce risk and minimize the loss of associated infrastructure (Nicholls and Leatherman 1996). In the Pacific islands, this could mean abandoning some low-level islands or abandoning low-level areas and moving to higher ground, if available on the same island. Accommodation suggests changing land use as water levels rise, such as raising buildings or changing to more salt-tolerant crops. Protection often uses constructed barriers to keep the sea away from coastlines (Nicholls and Leatherman 1996). Approaches can include hard structures such as seawalls and breakwaters or softer options such as the use of vegetation to stabilize beaches (Watson et al. 1996). Adding sand and stone to existing beaches or raising the height of some coastal villages may be useful in some places (Nicholls and Leatherman 1996). Precautionary approaches include the enforcement of enlarged building setbacks, land-use regulations, and building codes (Watson et al. 1996).

## What Are the Health Implications of Climate Variability and Change in Other Sectors?

Climate change will interact with and exacerbate other factors that contribute to the vulnerability of a particular region, as highlighted in the following examples.

Many small island states rely on a single source for their water supply, such as ground-water, rainwater, surface reservoirs, or shallow wells that draw from freshwater lenses just beneath the surface (Meehl 1996). These sources are climate sensitive, and changes in precipitation or rising sea levels will present challenges to public health. For example, the freshwater supplies of almost all Pacific islands are threatened during times of drought. Rising sea levels can result in salinity intrusion into the freshwater lens. Completely protecting water sources from adverse effects of climate variability and change may not be possible, but the management of water resources can be improved. Timely, site-specific, and reliable forecasts of impending droughts and floods that are rapidly disseminated to water resource managers and disaster planners are needed. Better management of the demand for water, water storage, and water loss prevention will help conserve supplies during times of droughts. More vigilant monitoring of water quality will be required.

Agriculture of subsistence food crops and crops for export may be adversely affected by changes in precipitation, rising temperatures causing heat stress to plants, salinization resulting from sea-level rise, and extreme weather and climate events, such as cyclones, floods, or droughts. For example, El Niño changes in 1991 and 1994 caused widespread rice crop failures in Indonesia (Buan et al. 1996). Approximately 50% of the rice and corn crop losses in the Philippines were due to climate variability. Decreases in agricultural productivity can obviously affect human health.

Coral reefs are an integral part of many island ecosystems. Coral reefs are likely to experience adverse effects from a range of climate change–related events such as increases in seawater temperature, sea-level rise, changes in storm patterns and coastal currents, changes in rainfall patterns, and additional pressures from nearby cities and settlements (Watson et al. 1996). Although coral reefs can recover from brief episodes of warmer water, prolonged periods (> 6 months) of increases in seawater temperature results in irreversible bleaching (Brown and Suharsono 1990). For example, the coral reefs that surround the Maldives play a role in the physical preservation of the islands and in fisheries, tourism, and local traditions and culture (WHO 2003). In 2000, tourism provided 33% of the Maldives’ GDP, and the fishing industry provided 6%. The reefs are the main attraction for tourism and provide fish for consumption and export as well as bait for the tuna fishery. Stress on the reefs originates from dredging, coral and sand mining, harbor construction, reclamation, construction of seawalls and jetties, and island-based pollution. Global sources of stress include climate change, El Niño conditions, ozone depletion, and sea-level rise. Stress to the reefs threatens fresh water and other natural resources, making island communities more vulnerable to natural disasters, as evidenced in the 2004 Asian tsunami.

## Recommendations

Because the health impacts of climate variability and change can be direct, indirect, multiple, simultaneous, and significant, governments, policy makers, decision makers, and resource managers in small island states are faced with major challenges. The capacity to undertake vulnerability assessments and develop adaptation policies and measures requires information on the health impacts of climate variability and change at the local and regional level; this information must include a comprehensive perspective from multiple sectors.

The workshops identified the following recommendations for improving the capacity of the health sector to anticipate and prepare for climate variability and change in small island states.

### High-priority research

- Expand knowledge of climate-sensitive diseases of importance in small island states through national and regional research. Research is particularly needed for diseases where information is limited, such as for skin, respiratory, and waterborne diseases. Identify and map locations, hazards, and communities especially vulnerable to climate variability and change, including sea-level rise, taking a holistic, cross-sectoral view. Establish verifiable links between ENSO, extreme weather events, climate variability, and health consequences in small island states.
- Conduct basic entomologic research, including the distribution of vector species, habitats, and biting habits, and their responses to climate variability.
- Improve understanding of the complex relationship between the risks posed by climate variability and change, and by other factors that influence population health. Expand knowledge of the social, cultural, and economic factors that modify vulnerability.
- Develop and evaluate indicators of the potential health impacts of climate variability and change at the national and regional level, incorporating environmental, social, and human dimensions.
- Understand the links between climate and other sectors, such as agriculture and water supply, and how these could affect health.

### Capacity building (including institutional needs)

- Develop institutional arrangements for knowledge sharing at national, regional, and international levels, including identification of regional centers of excellence; promote national and regional interdisciplinary working groups to study the impact of climate variability and change on health; and develop effective mechanisms for information sharing
- Improve education and training through workshops, follow-on networking, and structured training at local, national, and regional levels.
- Encourage programs of action and partnerships between public and private sectors, including business and nongovernmental organizations.
- Transfer knowledge of adaptation options to countries with similar climate/health concerns.

### Advocacy and community awareness of the potential impact of climate variability and change on human health

- Build awareness of the potential impact of climate variability and change in small island states across the full range of stakeholders, including local communities and the media; educate young people and medical/health professionals about climate/health links through school and university curricula; and work with policy makers to enhance awareness of climate variability and change and to catalyze discussions at national and regional levels.
- Incorporate a consideration of climate/health interactions in planned and ongoing development programs and include health aspects in global, regional, and local environmental and disaster-management planning. Advocate for integrated policy development across sectors to take account of the effects of climate variability and change on health.
- Develop advocacy messages in brief, nontechnical language for decision makers and policy audiences; ensure that advocacy messages reach key decision makers and policy audiences through appropriate channels and formats.

### Adaptation strategies, policies, and measures to reduce projected impacts

- Develop, improve, implement, and monitor early-warning systems.
- Monitor and evaluate the effectiveness of other public health interventions implemented to address the health impact of climate variability and change, including integrated pest management.
- Develop long-term adaptive strategies for sea-level rise on the basis of an understanding of current coping efforts and national development priorities.
- Assess the costs and benefits of intervention options.

### Data needs

- Collect more valid and comprehensive health, meteorologic, environmental, and socioeconomic data at appropriate local, regional, and temporal scales for research, program planning, and advocacy. Conduct inventories of existing data, identify current data gaps, and develop strategies to fill these gaps. Improve health surveillance systems to allow assessment of the impact of climate variability and change on health.
- Establish better data-management systems, programs, and practices, including the establishment of data-quality standards and best practices.
- Identify, engage, and enhance appropriate national and regional institutions for handling and analyzing data.
- Encourage fuller use of available data through regional and national capacity building (human resources, information technology, etc.). Improve sharing and timely access to relevant data sets.

### Climate forecasts

- Develop and improve national and regional forecasting capacity.
- Create partnerships between climate/meteorology and public health/medical specialists to improve awareness of the use and uses of climate forecast information. Provide user-friendly forecasts and applications information at national and regional levels.
- Facilitate communication between the public health/medical communities and national meteorologic and hydrologic services, as well as other relevant agencies and organizations.

### Resources needs

- Improve international, national, and regional facilities and funding for capacity building, interdisciplinary research, and regional/ national assessments. Establish programs with WHO, World Meteorological Organization, and United Nations Environment Programme in collaboration with other relevant agencies to provide country assistance in conducting vulnerability and adaptation assessments. Ensure that adequate funding is made available for priority research on climate and health from both the public and private sectors.
- Mobilize funding through all available mechanisms, including the Programme of Action for the Sustainable Development of Small Island Developing States (Barbados Programme of Action), the United Nations Framework Convention on Climate Change, the Global Environment Facility, the United Nations Convention on Biological Diversity, and the United Nations Convention to Combat Desertification.

## Conclusions

As stated in the Madang Commitment towards Healthy Islands (WHO 2001), small island states are places where children are nurtured in body and mind, environments invite learning and leisure, people work and age with dignity, the ecologic balance is a source of pride, and the ocean is protected (WHO 2001). Increasing understanding of the potential health impacts of climate variability and change in small island states and building capacity to cope with climate change through adaptation planning will help ensure that small island states will be prepared to deal with what the future does bring.

## Figures and Tables

**Table 1:** Demographic, health, and economic indicators in small island states.

	Population				Expectation of lost healthy years at birth (years)		Probability of dying per 1,000 (under 5 years of age )			
Small island state	Thousands	0–14 years of age (%)	Urban (%)	HALE (at birth)	Males	Females	Males	Females	Annual growth rate (%),1992–2002	GDP per capita (US$)
Africa										
Cape Verde	454	39.0	62	60.8	7.9	10.0	42	30	2.2	1,259
Comoros	747	43.0	33	54.6	7.8	9.6	80	72	3.0	278
Mauritius	1,210	25.6	43	62.4	8.1	10.9	20	14	1.1	3,779
Sao Tome and Principe	157	47.0	47	54.4	7.5	9.0	80	82	2.6	312
Seychelles	80	39.0	64	61.2	9.6	12.3	15	10	1.0	7,850
Asia and the Pacific										
Bahrain	709	27.0	92	64.3	7.9	10.1	13	10	3.0	12,012
Cook Islands	18	NR	59	61.6	8.6	11.5	21	19	−0.2	4,388
Fiji	831	33.0	49	58.8	7.7	9.7	30	27	1.2	2,046
Kiribati	87	NR	39	54.0	9.5	11.0	80	69	1.5	468
Maldives	309	37.8	27	57.8	7.5	9.0	38	43	3.0	1,806
Marshall Islands	52	49.0	72	54.8	7.2	8.9	46	36	1.3	1,938
Micronesia, Federated States of	108	44.0	28	57.7	7.9	9.6	63	51	0.7	2,215
Nauru	13	NR	100	55.1	6.9	9.0	18	12	2.5	2,500
Niue	2	NR	NR	60.4	8.6	11.3	38	24	−1.2	
Palau	20	NR	72	59.6	7.7	10.4	24	22	2.3	6,179
Papua New Guinea	5,586	40.0	13	51.9	7.0	9.1	98	92	2.6	545
Samoa	176	41.0	22	59.7	7.6	9.4	27	21	0.8	1,402
Singapore	4,183	21.2	100	70.1	8.6	10.4	4	3	2.8	20,544
Solomon Islands	463	45.0	20	56.2	8.3	10.3	86	75	3.2	760
Tonga	103	39.0	38	61.8	8.2	9.6	23	15	0.3	1,284
Tuvalu	10	41.0	52	53.0	7.0	8.3	72	56	1.4	1,342
Vanuatu	207	42.0	20	58.9	8.0	9.8	40	40	2.7	1,085
Europe										
Cyprus	796	22.0	57	67.6	8.8	10.6	7	7	1.2	11,504
Malta	393	19.0	91	71.0	6.2	8.0	7	6	0.7	9,245
Latin America and the Caribbean										
Antigua and Barbuda	73	28.0	37	61.9	8.9	10.3	22	18	1.2	10,204
Bahamas	310	29.0	89	63.3	8.1	9.5	13	11	1.5	14,856
Barbados	269	20.0	50	65.6	7.6	9.8	17	15	0.4	9,255
Cuba	11,271	20.0	75	68.3	7.9	9.8	8	7	0.4	2,545
Dominica	78	33.0	71	63.7	9.1	10.2	13	14	0.7	3,367
Dominican Republic	8,616	32.0	65	59.6	7.7	9.6	37	30	1.7	2,500
Grenada	80	NR	38	59.2	7.5	8.9	25	21	–0.5	4,682
Haiti	8,218	39.0	36	43.8	5.6	6.9	138	128	1.4	431
Jamaica	2,627	31.0	56	65.1	6.9	8.6	16	14	0.9	2,990
Saint Kitts and Nevis	42	30.6	34	61.5	8.7	9.1	20	24	0.0	6,396
Saint Lucia	148	32.0	38	62.7	8.6	10.2	14	15	0.9	4,994
Saint Vincent and the Grenadines	119	37.0	55	61.0	7.9	9.8	25	20	0.6	1,940
Trinidad and Tobago	1,298	23.0	74	62.0	7.3	8.6	24	18	0.5	6,817

NR, not reported. Data from United Nations (2003) and WHO (2002).

**Table 2:** Environment and climate indicators in small island states.

			Annual temperature (ºC)		
Small island state	CO_2_ emissions (megatons)	Energy consumption per capita (kg oil equivalent)	Minimum	Maximum	Annual precipitation (mm)
Africa					
Cape Verde	121	108	23.5^a^	29.3	70
Comoros	66	38	21.2	29.5	2,700
Mauritius	1,704	680	20.2	26.9	1,793
Sao Tome and Principe	77	226	23.3^b^	28.6^b^	1,040^b^^,^^c^
Seychelles	198	893	23.9	31.0	2,172
Asia and the Pacific					
Bahrain	14,847	12,889	14.1	38.0	72
Cook Islands	22	578	20.7^b^	26.7^b^	2,103^b^
Fiji	755	323	20.4	31.0	3,040
Kiribati	22	100	27.6	28.1	100
Maldives	304	557	25.1	31.5	1,951
Marshall Islands	NR	NR	26.7	27.7	2,407
Micronesia, Federated States of	141	NR	23.4^b^	31.2^b^	469^b^
Nauru	139	3,666	25.0^b^	29.9^b^	2,236
Niue	NR				
Palau	234	4,404	24.2^b^	31.0^b^	3,746
Papua New Guinea	2,451	188	25.4	27.7	1,150
Samoa	132	287	24.4^b^	29.9^b^	2,928
Singapore	35,634	3,873	24.9	31.6	2,191
Solomon Islands	161	128	22.3	30.7	3,290
Tonga	121	406	20.2^b^	26.8^b^	1,610^b^
Tuvalu	5	NR	NR	NR	NR
Vanuatu	62	138	21.5	28.2	2,222
Europe					
Cyprus	5,456	2,365	7.3	32.3	320
Malta	1,759	2,841	9.2	30.7	553
Latin America and the Caribbean					
Antigua and Barbuda	337	1,799	23.9	29.6	1,052
Bahamas	1,740	1,994	16.7	31.8	1,360
Barbados	898	1,438	25.1	27.1	1,273
Cuba	25,113	581	18.6	31.6	1,189
Dominica	81	419	21.6	30.5	654
Dominican Republic	13,224	847	19.6	31.5	1,448
Grenada	183	707	25.1^b^	29.3^b^	1,359^b^
Haiti	1,389	63	NR	NR	NR
Jamaica	10,728	1,301	22.9	31.4	813
Saint Kitts and Nevis	103	807	25.1^b^	29.3^b^	NR
Saint Lucia	198	741	25.9^b^	29.1^b^	NR
Saint Vincent and the Grenadines	161	505	NR	NR	NR
Trinidad and Tobago	21,966	8,084	23.2	31.8	1,714

NR, not reported. Data from United Nations (2003) and WHO (2002).

a Average mean temperature.

b Data from The Weather Channel (2004).

c Data missing.
